# Case Report: A gambling-related suicide in rural Malawi

**DOI:** 10.12688/wellcomeopenres.17333.1

**Published:** 2021-11-11

**Authors:** Junious Mabo Sichali, Albert Dube, Lackson Kachiwanda, Heather Wardle, Amelia C Crampin, Christopher Bunn

**Affiliations:** 1 Malawi Epidemiology and Intervention Research Unit, Lilongwe, Malawi; 2 Social and Political Sciences, University of Glasgow, Glasgow, Scotland, G120QQ, UK; 3 Institute of Health and Wellbeing, University of Glasgow, Glasgow, Scotland, G120QQ, UK

**Keywords:** Gambling, Betting, Suicide, Malawi, Mental Health

## Abstract

Background

As in many other countries across sub-Saharan Africa, Malawi’s commercial gambling sector has grown considerably in recent years. Driven by the widespread availability of internet through mobile devices, the industry has penetrated both urban and rural settings. In Malawi the model commonly implemented by gambling companies is similar to that used by mobile phone operators. Agents equipped with cellular devices connect to providers’ servers to place wagers for customers and print receipts using simple printers attached to their devices. This has produced lucrative returns for providers. While increasing attention is being paid to this trend, most research focusses on sports betting and there is a deficit of papers that document gambling-related harms.

Methods

Here we present a narrative case report of a 16-year-old boy, ‘Wati’ (pseudonym), who lived in rural Malawi and took his own life after gambling and losing money that did not belong to him. As his community is part of a demographic surveillance site, a verbal autopsy was conducted, later supplemented with interviews with Wati’s close friend and uncle, to whom his mother referred us. We triangulated data from these three sources to create a narrative case report of Wati’s suicide and its relationship to his gambling practices.

Results

We found that the gambling harms leading up to Wati’s suicide were recurrent, that his gambling practices were diverse (lottery, football betting, digital games and cards) and that signs of distress were apparent before his suicide.

Conclusions

From this case report, we learn that underage individuals participate in gambling in Malawi, can develop harmful habits and that their gambling is not confined to sports betting. We also learn that there is a lack of accessible services for people who develop harmful gambling practices. Wati could have benefited from such services and they may have saved his life.

## Introduction

Over the last decade, commercialised and massified forms of gambling have been introduced across sub-Saharan Africa (SSA) in the 40 territories where gambling is permitted ^1^. Much of the growth has been fueled by high levels of engagement with European football, which betting companies have capitalized on ^2– 11^. Some have suggested that this industry expansion is comparable to the way in which tobacco companies shifted activities towards the territories with less stringent regulation after the introduction of tobacco control and that it risks public health ^1^.

A growing body of research has documented how this growth is being driven by young people in the SSA region and integrated into their everyday lives ^12, 13^. This research has noted that unemployment and lack of opportunity are important drivers of gambling engagement among young people ^2, 9, 14^, that it can be associated with alcohol consumption ^8^, and that it can provide a community and sense of social belonging ^7, 15^. The evidence also suggests that for some young people in SSA, gambling is form of escapism ^16^ which provides entertainment ^17^.

While motivations for and the cultures of youth gambling have been the focus of much of the burgeoning literature, few studies have described the gambling related harms that youth gambling engenders. A recent systematic review noted this dearth of evidence, a lack of standardized tools and differences in populations, arguing for the need to generate more systematic and comparable evidence from across the SSA region ^13^. The review did, however, note high levels of lifetime participation in gambling in all three regions of SSA, ranging from 57%–73% of the populations studied. The exception to this was Malawi, where lifetime participation was reported to be 13% ^18^. However, the Malawi study was conducted before the widespread introduction of accessible gambling providers, which occurred in 2015.

Since 2015, Malawi has seen a rapid growth in gambling participation as well as gross revenue realised ^3^. This growth has primarily been driven by the introduction of sports betting products by Premier Bet. Premier Bet was launched by Editec, a European company with offices in the United Kingdom, France, Romania, Malta and Sweden and currently operates in 15 SSA countries ^19^. Its sports betting products are made widely available by mobile internet technologies and road-side franchise-based outlets, which enlist local community members to sell these products using simple tablets and receipt printers ^2, 3^. The company also make extensive use of print and other media to advertise their products, with company representatives on record positioning gambling as a potential income, in the context of high unemployment ^3^. With mobile internet technologies, anyone with a smartphone can access the Premier Bet website to open an account, place a bet and pay using mobile money services. For those without a smartphone, services can be accessed via road-side franchise-based outlets or Premier Bet’s betting shops, which are available in almost all cities, towns and village trading centers in Malawi.

**Figure 1.**
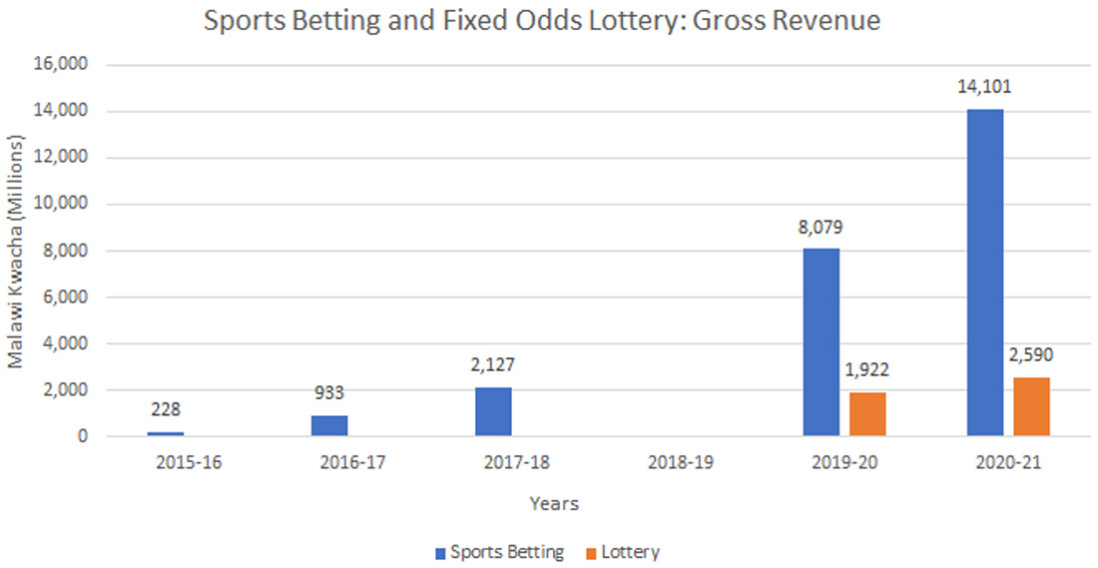
Gross Revenue from sports betting and fixed odds lottery markets in Malawi 2015–2021.

The Malawi Gaming Board’s (MGB) annual reports provide evidence of the impact the introduction of Premier Bet’s products have had on the market ^20– 22^. As we see in Figure 1, gross gambling revenue from sports betting in Malawi has risen steeply, since its introduction in 2015, with Premier Bet initially holding a 99% market share, which gradually reduced to 89% with entry of Supapesa and World Star Betting into the market.

The Fixed Odds Lottery (FOL) market was established in 2019, with Premier Bet granted an exclusive license for this category of product and granted the status of a ‘National Lottery’. The FOL products the company offers in Malawi are diverse and the primary FOL is quite different to lottery products in the Global North. The primary FOL offered by Premier Bet is Premier Loto 5/90, which is a 90 ball lottery drawn four times a day, five days week, producing 20 draws per week ^23^. Alongside this FOL product, Premier Bet offer a range of instant products including an instant lottery and digital scratch cards ^24^. These products include draws which take place as often as every five minutes and algorithm-based instant play options. While the growth rate for these FOL products has not been as rapid as in Premier Bet’s sports betting market, data from the MGB in Figure 1 demonstrate strong returns in the first year of operation and growth in the second.

To our knowledge, only one study in Malawi has explored the impact of the introduction of widespread commercial gambling in Malawi. The study examined the experiences of young male sports bettors in Lilongwe, finding that their households diets, hygiene, inter-personal relationships and cognitive resources were negatively affected ^2^. No work has been done to document or explore suicidality in Malawi in relation to Gambling, yet we know from work elsewhere in the world that gambling harms are strongly associated with suicide and suicide attempts ^25– 28^.

Here, we add to the limited literature on gambling-related harms in Malawi and the wider SSA region by offering a case report of a gambling-related suicide which occurred in a rural area of the northern region of the country. We use verbal autopsy and narrative case report methods to describe the events surrounding the suicide. In the discussion section, we consider what policy and service responses may be able to prevent the tragic events we describe.

## Methods

This case report was based within the rural Karonga Health and Demographic Surveillance Site (HDSS), established in 2002 by the Malawi Epidemiology and Intervention Research Unit (MEIRU, formerly Karonga Prevention Study) in Northern Malawi. Within the HDSS, censuses of the population of 49,597 individuals are conducted annually, collecting data on demographic, social and health indicators. Through a network of key informants that live in the communities, there is also continuous reporting on migration, births, and deaths.

When deaths are reported within the Karonga HDSS, a verbal autopsy (VA) is conducted two weeks after the death. Although the question set is similar to, and consistent with, the World Health Organization’s standard tool, the MEIRU VA (verbal autopsy) tool, includes a narrative of circumstances surrounding the death based on informant(s) interview ^29^. The case report we present here utilised this standard procedure, with a fieldworker (LK) visiting the household of the deceased and interviewing his mother. The VA form was completed and cause of death assigned by two independent clinicians, as per standard MEIRU procedures. On discovery of the gambling-related nature of the death, an expanded verbal autopsy procedure was conducted with additional brief interviews conducted with the deceased’s uncle and best friend.

Data from the three interviewees were transcribed in note form by the fieldworker, who, with permission from the interviewees, shared them with two social scientists (JMS and CB) experienced in qualitative methods and who study gambling in Malawi. The two social scientists read the transcriptions, taking notes to establish the sequence of events that led up to the suicide, triangulating the different accounts they contained for points of confirmation and discrepancy using Microsoft Word (v16). Demographic data relating to the deceased is linked in the HDSS database. Once a sequence of events was established, the social scientists wrote this up as a narrative case report, a method which is well suited to exploring specific social problems and the sequence of events that led to the problem, in context ^30^.

## Ethical approval

Ethical approval for the HDSS was granted by the National Health Sciences Research Committee (NHSRC) (protocol number #20/11/2641, 29/01/2021), and by the London School of Hygiene and Tropical Medicine (LSHTM) (protocol number #5081, 20/02/2007). All participants gave written informed consent to participate. The mother of the deceased gave written consent for this case report to be published, after having it read aloud to her.

## Case report

In June 2021 the people of Muzi Withu (pseudonym) in the Karonga district of Malawi, woke up to news of the death, by suicide, of a 16-year-old black African boy, Wati (pseudonym). Wati’s suicide occurred after gambling and losing K22,000 (approximately £20), playing lottery games offered by Premier Bet. The demographic data relating to Wati suggest that Wati’s early childhood was typical of his peers. He fell behind in his education and at aged 16 was still studying at primary school. In the period leading to his death he was living with his mother, but frequently spent extended periods living with his uncle at the local trading center, and had no regular employment. Wati’s medical history indicated that he had diagnoses of asthma and epilepsy, however his health passport indicated that he did not have any symptoms of these conditions during the period leading to his death. He had tested negative for HIV and he was not known to drink alcohol or smoke.

The three narratives (mother, uncle and friend) suggest that Wati began gambling between one and two years prior to his death. His mother was aware that he was betting on lottery products, but his best friend and uncle were aware that he was also engaged in sports betting, electronic gaming machines and informal card games. Wati’s mother suspected that he was gambling daily, and both his friend and uncle reiterated that he was gambling frequently, with his friend suggesting that Wati was ‘addicted’ to gambling. His mother believed that his son decided to take his own life due to worries that he had during the period leading to his death. This information was also corroborated by the uncle and his best friend who said that he had worries relating to his routine gambling losses as well as how he was going to repay the people that he owed money to.

During the interview with Wati’s uncle, he revealed that within the last year he had to settle a debt of K84,000 (~£80, which could be equivalent to a monthly salary in this setting) with Wati’s former employer. Wati had been operating a motorcycle taxi and received customer payments amounting to this sum, which he failed to give to his employer. Instead, Wati’s uncle explained, he spent the money on betting.

The verbal autopsy narratives that we collected indicated that the boy was friends with a butcher who had asked Wati to go and collect K22,000 from a customer who had bought meat. After collecting the cash payment, Wati took the money to the local trading center and spent it over the course of one weekend, playing lottery games at the local Premier Bet outlet. When subsequently approached by the butcher who asked for the money he was sent to collect, Wati lied and said that he had given it to his brother for safe keeping. This annoyed Wati’s brother who attacked him. Meanwhile the butcher wanted to recover his lost money and seized Wati’s laptop bag (containing his notebooks and a food container), in an attempt to do so.

During the days leading up to his death, his uncle reported noticing a change in Wati’s behaviour, observing that Wati was looking confused and unsettled such that he was rarely found at home, presumably to avoid meeting those to whom he owed money, and was always betting. His best friend told us that following the altercation with his brother and the confiscation of his bag, Wati was reported missing from home for 18 hours, from 11am up to 5am the following day when he was discovered dead in the nearby woods. His body was found by a woman from the same village who woke up early in the morning to go to the lake to buy fish. 

## Discussion

Wati’s story is tragic and was avoidable. Even though his medical history indicates that he had comorbidities, his medical records note that at the time of his death he had no symptoms of illness. His story raises significant questions for the public health community, policy makers, regulators, service providers and Malawian society at large. At just 16 years of age, Wati stole money from informal employers to fund his gambling on two occasions, lied to conceal his gambling, lost a job and damaged his relationship with his brother due to his gambling, relied on his uncle to cover a significant gambling debt and exhibited signs of distress after his loss. Wati’s best friend labelled him ‘addicted’ to gambling, with a notable preference for lottery products that follow an intense or instant pattern of play. It is therefore likely that Wati’s gambling practices would have be deemed ‘disordered’ by a psychiatrist applying criteria set out in the fifth edition of Diagnostic and Statistical Manual of Mental Disorders, although the severity of his situation will never be known ^31^.

What is known, is that Wati’s suicide followed and was linked to a range of harms that are widely identified in literature from high-income countries: financial, relationship and family, criminal and mental health ^32^. These harms were not confined to Wati, but were also experienced by his family, friends and informal employers, as well as the wider community which grieved his lost life; another characteristic of gambling-related harms identified by researchers from high-income countries ^33^. Finally, it is clear that Wati’s case fits literature on suicidality and gambling from high-income countries, which has demonstrated associations between problem gambling and suicide attempts ^26^.

While there has been an historical tendency towards pathologizing and individualizing gambling problems, scholarship and public discourse is increasingly shifting towards a public health perspective on gambling ^33, 34^. This perspective emphasizes the interaction between individuals and their complex social environments ^35^, the need to prevent the entrenchment of health inequalities via the gambling harms experienced by the most disadvantaged ^32^, and the need to regulate the ‘availability, licensing, advertising, and price of products’ ^33^.

In the Malawian context, the prevailing paradigm for addressing harms, as indicated in the MGB’s annual reports ^21^, is the ‘responsible gambling’ model. This places the burden of responsibility, and the blame for negative outcomes, on individual gamblers ^34^. However, Wati’s case demonstrates the flaws in this approach: he was allowed to gamble despite being underage, which is a structural and regulatory failure; he was exposed to gambling products which were readily available, cheap and widely advertised, which are consequences of the social, media and policy environments in which he lived ^2, 3^; and no formal services were accessible to him or his family following his first loss of stolen money, demonstrating the insufficiency of the support structures for those whose gambling becomes harmful. While we do not wish to reduce Wati’s case to these social factors, it is clear that it cannot be understood without reference to them nor reduced to an individual pathology.

The case report we have presented has both strengths and limitations. The narrative case report is based on triangulated data from two of Wati’s family members and his best friend, ensuring that multiple perspectives on his suicide were obtained. In addition to this, the demographic data we present are robust due to Wati’s participation in the HDSS. However, the case report would have been improved by more extensive interviewing of those who spent time with Wati during his last days.

## Conclusion

In conclusion, Wati’s case challenges us to improve regulatory enforcement in Malawi, to provide better public health approaches to mitigating gambling harms, including through provision of referral services with wide reach and few barriers to access, and to recognise the severity of the failure to manage the industry well: unnecessary loss of life that devastates families and communities.

## Consent

All participants gave informed consent before data was collected. The mother of the deceased gave written consent for this case report to be published, after having it read aloud to her. 

## Data availability

All data underlying the results are available as part of the article and no additional source data are required.
