# Supplementary material for: Case Report: A gambling-related suicide in rural Malawi
Source: Wellcome Open Res. 2021 Nov 11;6:308. [Version 1] doi: 10.12688/wellcomeopenres.17333.1 (PMC8609396; doi:10.12688/wellcomeopenres.17333.1)
Supplement: Peer review history

# Peer review: Reviewer response for version 1

**DOI:** 10.21956/wellcomeopenres.19163.r47058

**Published:** 2021-11-22

**Authors:** Manase Kudzai Chiweshe

**Affiliations:** 1 Faculty of Social Science, Community and Social Development, University of Zimbabwe, Harare, Zimbabwe; 2 Sociology Department, Rhodes University, Grahamstown, South Africa

The case study provides important insights into the negative impacts of sports betting in resource-constrained contexts. It highlights how gambling can have devastating effects in contexts where there is a lack of institutional support for punters. Rural Malawi thus provides nuances that highlight how young people can end up in fatal situations due to gambling. 

 The case is well constructed but I feel the background of rural Malawi and related context of poverty need to be more clearly spelt out to show how for youths such as 'Wati', gambling can seem like the only way out. The background to the case study, therefore, needs to provide more information on youths and the everyday challenges of poverty in rural Malawi, especially for young men.

Are enough details provided of any physical examination and diagnostic tests, treatment given and outcomes?

Yes

Is the case presented with sufficient detail to be useful for other practitioners?

Yes

Is sufficient discussion included of the importance of the findings and their relevance to future understanding of disease processes, diagnosis or treatment?

Yes

Is the background of the case’s history and progression described in sufficient detail?

Partly

Reviewer Expertise:

Sports and football studies in Africa

I confirm that I have read this submission and believe that I have an appropriate level of expertise to confirm that it is of an acceptable scientific standard.

# Peer review: Reviewer response for version 1

**DOI:** 10.21956/wellcomeopenres.19163.r47060

**Published:** 2021-11-15

**Authors:** Charles Livingstone

**Affiliations:** 1 School of Public Health and Preventive Medicine, Monash University, Melbourne, Vic, Australia

This is a significant article that describes an important aspect of the harms associated with gambling in a low income country. As the authors note, the growth of gambling revenue in many countries of Sub-Saharan Africa is occurring in an environment where familiarity with the harms of gambling may be low, which may undermine efforts to introduce effective regulation. Further, although the authors note the deficit of papers addressing gambling harms, it is also important to realise that assessing the impacts of gambling via a harms-based approach is relatively novel at a global level. The dominance of the 'responsible gambling' model has, as the authors also note, meant that the historical tendency for gambling harms to be identified as an individual responsibility has been, and in many cases continues to be, widespread. This is as much an issue for high and middle income countries as it is for low income countries.

 The methods adopted for this report are interesting and appropriate. The potential for this to become an important way of documenting a particularly tragic aspect of gambling harms is very high. I find the triangulation approach adopted to be very apt, and to add significantly to the identification of key issues and the overall integrity of the report. I am particularly impressed by the clarity of the report, and its potential to be utilised as a model for further reporting. The analytical consequences relating to failures of regulation and enforcement, the inadequacy and inappropriateness of the responsible gambling model, and the myth, apparently promoted by gambling operators, that gambling can enhance one's income, are all important. This approach has a great deal to add to the development of a critique of public policy and should in my view be adopted wherever possible.

 Certainly, the WHO's increasing interest in gambling and gambling disorder is increasingly focused on how to support low and middle income countries in effective and appropriate regulation, and case studies such as this can be an important tool to inform discussion and further research. I would suggest that some further aspects might be contemplated for further work. The first of these is the extent to which gambling related shame or stigma may be associated with the responsible gambling discourse, and also to suicidality (for example see my article, Livingstone & Rintoul 2021 ^1^) and the second to how best to address this, and other regulatory failures. Public Health England has recently produced a series of papers addressing gambling related harms and is finalising research into what interventions might be effective. The first set of papers is available at: https://www.gov.uk/government/publications/gambling-related-harms-evidence-review and the research into effectiveness of interventions is apparently forthcoming. Myself and colleagues also completed a review of interventions from cognate areas of harmful commodities with a history of public health interventions in 2019 (available at https://responsiblegambling.vic.gov.au/resources/publications/identifying-effective-policy-interventions-to-prevent-gambling-related-harm-640/). 

 The export of the harms of gambling into low and middle income countries is a matter of considerable concern. However, the documentation of harms is an important step in developing effective frameworks for their prevention and minimisation. For this reason, this article is of significance and importance.

Are enough details provided of any physical examination and diagnostic tests, treatment given and outcomes?

Yes

Is the case presented with sufficient detail to be useful for other practitioners?

Yes

Is sufficient discussion included of the importance of the findings and their relevance to future understanding of disease processes, diagnosis or treatment?

Yes

Is the background of the case’s history and progression described in sufficient detail?

Yes

Reviewer Expertise:

Gambling policy and regulation, harm prevention and minimisation, public health, gambling harm, social theory.

I confirm that I have read this submission and believe that I have an appropriate level of expertise to confirm that it is of an acceptable scientific standard.

## References

[1] : Gambling-related suicidality: stigma, shame, and neglect. *The Lancet Public Health* .2021;6(1) : 10.1016/S2468-2667(20)30257-7 e4-e5 10.1016/S2468-2667(20)30257-7 33417846
